# Survivorship-Reducing Effect of Propylene Glycol on Vector Mosquito Populations and Its Potential Use in Attractive Toxic Sugar Baits

**DOI:** 10.3390/insects13070595

**Published:** 2022-06-29

**Authors:** Heidi Pullmann Lindsley, Henry B. Lyons, Melissa Leon-Noreña, Ronald Jason Pitts

**Affiliations:** Department of Biology, Baylor University, Waco, TX 76798, USA; heidi_lindsley1@baylor.edu (H.P.L.); henry_lyons1@alumni.baylor.edu (H.B.L.); melissa_leonnorena1@alumni.baylor.edu (M.L.-N.)

**Keywords:** toxin, ATSB, GRAS, *Aedes aegypti*, *Aedes albopictus*, *Culex pipiens*, vector control, insecticide resistance

## Abstract

**Simple Summary:**

Mosquitoes (Diptera: Culicidae) spread disease and pose a significant risk to public health around the world. While there are currently many control measures available, many are typically unsafe for humans and other animals, and they are becoming less effective against mosquitoes. We tested a compound called propylene glycol (1,2 propanediol) for its toxicity to three species of mosquitoes that serve as vectors of human pathogens. Propylene glycol is a compound that the FDA has designated as generally regarded as safe (GRAS) for human consumption, meaning it is approved for use in everyday household products. Through a series of assays in which we fed mosquitoes propylene glycol, we found that this compound is highly toxic to all three mosquito species examined and can drastically reduce the survivorship of laboratory populations. Our results suggest that propylene glycol could be a safe and effective substance to be used in the context of attractive toxic sugar baits (ATSBs) as a means of controlling mosquitoes near human habitations.

**Abstract:**

Arthropod control mechanisms are a vital part of public health measures around the world as many insect species serve as vectors for devastating human diseases. *Aedes aegypti* (Linnaeus, 1762) is a widely distributed, medically important mosquito species that transmits viruses such as yellow fever, Dengue, and Zika. Many traditional control mechanisms have become less effective due to insecticide resistance or exhibit unwanted off-target effects, and, consequently, there is a need for novel solutions. The use of attractive toxic sugar baits (ATSBs) has increased in recent years, though the toxic elements are often harmful to humans and other vertebrates. Therefore, we are investigating propylene glycol, a substance that is generally regarded as safe (GRAS) for human consumption. Using a series of feeding assays, we found that propylene glycol is highly toxic to *Ae. aegypti* adults and a single day of exposure significantly reduces the survivorship of test populations compared with controls. The effects are more pronounced in males, drastically reducing their survivorship after one day of consumption. Additionally, the consumption of propylene glycol reduced the survivorship of two prominent disease vectors: *Aedes albopictus* (Skuse, 1894) and *Culex pipiens* (Linnaeus, 1758). These findings indicate that propylene glycol could be used as a safe and effective alternative to pesticides in an ATSB system.

## 1. Introduction

The control of invasive and dangerous arthropod vectors is a pressing goal in entomology. *Ae. aegypti*, commonly known as the yellow fever mosquito, is widely established in the tropics, subtropics, and temperate zones and will likely spread to new regions as climate change and global trade continue to shape our world [1,2,3,4,5]. In addition to yellow fever, *Ae. aegypti* females are vectors for other serious flaviviruses, such as Dengue and Zika [6,7]. These diseases impose a heavy burden in Asia, Africa, and South America, with a prominent risk of viral outbreaks every year [8,9]. Some studies have estimated that approximately 390 million cases of Dengue occur annually [10] and while outbreaks of yellow fever and Zika are less prominent, epidemics are still possible [11]. Another mosquito of public health concern is *Ae. albopictus*, a highly invasive species that is competent for transmitting numerous viruses and is a known vector for Chikungunya [12]. Lastly, *Cx. pipiens* is a competent vector for a number of arboviruses, including Japanese encephalitis virus and West Nile virus [13]. Collectively, these species are responsible for a significant portion of vector-borne disease transmission worldwide. Their encroachment into new regions, therefore, poses a significant public health risk [14,15,16].

One control measure that has been proven effective in the management of localized mosquito populations is the use of attractive toxic sugar baits (ATSBs) [17,18,19,20]. While conventional control methods have led to problems, such as insecticide resistance and off-target effects, ATSBs are designed to have more specific effects on the desired species and to minimize the non-lethal exposure to insecticides [21,22]. These are devices that combine an attractant to lure insets with a toxin to control adult mosquito populations [23,24,25]. However, one remaining issue with ATSBs is that the insecticides commonly used in the system are potentially harmful to humans and continue to contribute to insecticide resistance [26,27,28,29,30,31]. Therefore, as *Ae. aegypti* mosquitoes threaten to spread disease throughout the world, novel insecticides that are safe for humans are increasingly important [32,33]. An ideal insecticide substitute would be safe to humans, have a different mode of action than the most frequently used commercially available insecticides, lead to population decrease, and disrupt disease cycles [34,35,36].

Since 1958, the FDA has curated a list of food and cosmetic additives that are generally regarded as safe (GRAS). These substances are additives that no longer require formal FDA review, as their safety for humans has long been established by qualified experts [37]. The compounds are typically intended for the preservation of packaged food or for the postharvest management of produce. While these substances are safe for humans, it has been shown that some GRAS compounds have negative effects on arthropods [38]. This paper explores one GRAS substance, propylene glycol, as a potential substitute for other insecticides [39,40,41]. Propylene glycol, a food additive used as a solvent and preservative for food colors and flavors, was selected as an initial GRAS compound to test because it is almost completely without odor or flavor, readily mixes with water, and is relatively accessible. Some more specific uses of propylene glycol include as a pharmaceutical solvent for oral, topic, and injected medicines, as well in personal care products, such as toothpaste and hand sanitizer, in addition to in cleaning products [42,43]. However, some studies have shown that propylene glycol can cause contact dermatitis in a small percent of people and that rapid, large intravenous doses can be toxic [44,45,46,47]. While these health effects necessitate more research, the consensus is that propylene glycol is safe for human use and consumption [48].

For this study, we assessed *Ae. aegypti* adult survivorship over time when challenged with propylene glycol in the context of sucrose solutions. In our initial tests, we examined the effects of three concentrations of propylene glycol under two distinct feeding paradigms: ad libitum and 24 h availability. Daily survivorship is one important factor that influences population dynamics, and it is the only one that was considered in this study [49]. Other factors, such as birth and death rates, immigration, and emigration, were not examined, as our study was limited to laboratory conditions. We also assessed the effect of propylene glycol on *Ae. albopictus* and *Cx. pipiens* adults to explore its potential toxicity to other prominent disease vectors. We established that even at 5%, the lowest concentration tested, propylene glycol could eliminate an entire population in seven days when offered ad libitum. At higher concentrations, the effect was more pronounced. Moreover, populations of all three species were significantly reduced when propylene glycol was presented for only 24 h. Additionally, we observed greater lethality in *Ae. albopictus* and *Cx. pipiens*, indicating that propylene glycol can be used as an insecticide for a variety of mosquito vectors.

## 2. Materials and Methods

*Mosquito Rearing: Aedes aegypti* and *Aedes albopictus* larvae were collected from standing water in small outdoor containers at a private residence in McLennan County, Texas, near the city of McGregor. Larvae were reared to adulthood, and species identities were confirmed by adult morphological characters. Colonies of the “McGregor” strains were established in the laboratory and maintained under standard conditions: 27 °C, 70% RH, 12:12 LD, 5% sucrose (*w/v*) in distilled water ad libitum. Females were fed defibrinated sheep blood (Hemostat Laboratories, Dixon, CA, USA) using a membrane feeding system (Hemotek Ltd., Blackburn, UK). Lab strains of *Culex pipiens*, which were originally collected and colonized at Ohio State University, were provided as eggs by the laboratory of Dr. Cheolho Sim at Baylor University. They were maintained under the same conditions as the *Aedes* species.

*Ad libitum Trials*: At pupal stage, mosquitoes were divided into cages (BugDorm-1; MegaView Sci. Co. Ltd., Taiwan) of approximately 50 individuals and provided with 25 mL of 5% sucrose solution (*w/v*) in a glass bottle with a cotton wick extending approximately one inch out of the bottle. The number of mosquitoes used was dependent upon the hatch rate and survivorship of the larvae to adulthood. Exact numbers per trial are provided in supplemental data. Seventy-two hours post-eclosion, the sucrose solution was removed from the cages, and the adult mosquitoes were starved for twenty-four hours. Following the starvation period, a solution of 5% sucrose plus 5%, 7.5%, or 10% propylene glycol (*w/v*; Fisher Scientific, CAS # 57-55-6) was colored blue with one milligram of powdered dye (Acid Blue 9, TCI America, CAS # 3844-45-9). Separate studies were conducted to verify the innocuous nature of the dye (data not shown). Cages were visually inspected every 24 h for 7 days. Mortality was determined by observing mosquitoes lying on the bottom of the cage, unmoving, with legs pointing upward and by the lack of response upon prodding. Expired mosquitoes were removed from the cage, sorted by sex, checked for evidence of feeding (presence of blue dye in the abdomen), and counted. At the end of the 7-day trial period, the remaining mosquitoes were sorted by sex and counted. Trials were carried out in three biological replicates. Control cages were provided with a 5% sucrose solution, dyed blue, and prepared in the same fashion described above.

*24-Hour Trials*: Trials were initiated as described above. Mortality was assessed after 24 h, and the toxin was removed and replaced with a 5% sucrose solution, colored blue. Mortality was assessed every 24 h for a total of 7 days. At the end of the 7-day trial period, the remaining mosquitoes were sorted by sex and counted. Trials were carried out in three biological replicates.

*Statistical Analysis*: GraphPad Prism 9 was used for analysis. Kaplan–Meier curves were plotted to assess population survivorship, and significance was determined using a log-rank (Mantel–Cox) test with a post hoc analysis and a Bonferroni correction [50].

## 3. Results

### 3.1. Propylene Glycol Reduces Survivorship of Ae. aegypti Mosquitoes

**Figure 1:**
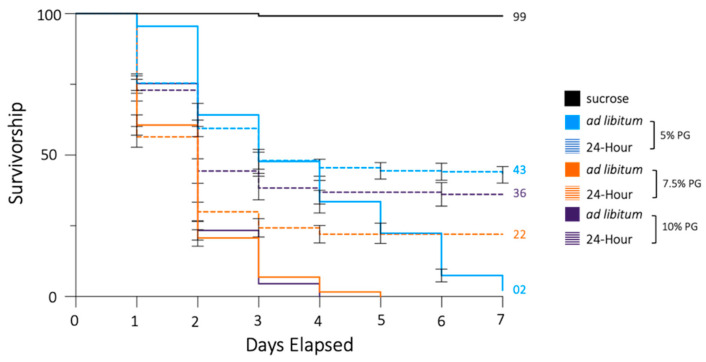
Propylene glycol reduces the daily survivorship of *Ae. aegypti mosquitoes.* Day 0 indicates the day propylene glycol solutions were added. A Kaplan–Meier curve demonstrates the decline in mosquito survivorship over the course of 7 days. Populations fed on sucrose, sucrose + 5% propylene glycol, sucrose + 7.5% propylene glycol, or sucrose + 10% propylene glycol ad libitum or for a 24 h period, at which point the propylene glycol solutions were replaced with 5% sucrose. Percentage of the population surviving at day 7 is provided when applicable. Error bars indicate SE of the mean.

Our study indicates that propylene glycol reduces the daily survivorship of *Ae. aegypti* adult mosquitoes. All the populations that fed on propylene glycol +5% sucrose at any concentration ad libitum had significantly reduced survivorship when compared to the control groups that fed on 5% sucrose only (Mantel–Cox post hoc Bonferroni correction *p* < 0.0001). The mosquitoes that fed ad libitum on 7.5% propylene glycol + sucrose or 10% propylene glycol + sucrose had a lower daily survivorship than the mosquitoes that fed on 5% propylene glycol + sucrose (*p* < 0.0001), though there was no significant difference between the groups at the two higher concentrations (Figure 1).

Additionally, there was a reduced daily survivorship between the groups that fed on any concentration of propylene glycol + sucrose for 24 h followed by 6 days of sucrose only, and those that fed on sucrose only for 7 days (*p* < 0.0001), and the survivorship differed between the three concentrations (Figure 1). The mosquitoes that fed on 7.5% PG + sucrose for 24 h had a lower survivorship than the 24 h 5% propylene glycol + sucrose group (*p* < 0.0001), but there was no statistical distinction between the groups that fed for 24 h on 5% propylene glycol + sucrose and 10% propylene glycol + sucrose, or between the two higher concentrations. Lastly, regardless of concentration, the daily survivorship of the adults that fed ad libitum was lower than that of those that only fed for 24 h (*p* < 0.0001). We found that the sex-specific effects were the same across concentrations (Figure S1).

### 3.2. PG Has Sex-Specific Effects on Ae. aegypti Mosquitoes

**Figure 2:**
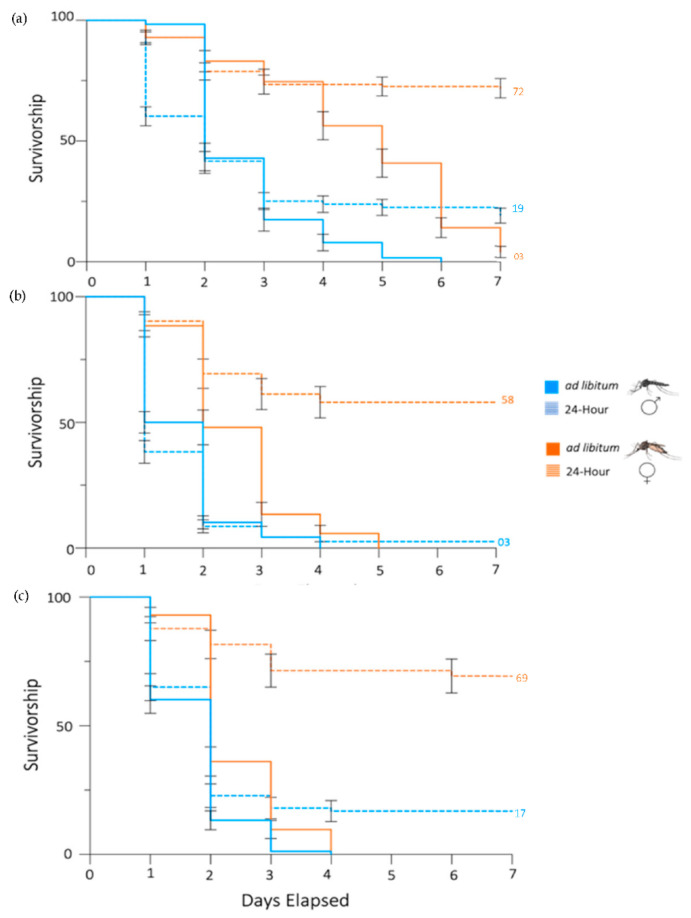
Propylene glycol reduces the daily survivorship of male and female *Ae. aegypti* mosquitoes. A Kaplan–Meier curve demonstrates the decline in mosquito survivorship over the course of 7 days: (**a**) 5% sucrose + 5% propylene glycol, (**b**) 5% sucrose + 7.5% propylene glycol, or (**c**) 5% sucrose + 10% propylene glycol ad libitum or for a 24 h period, at which point the propylene glycol solutions were replaced with 5% sucrose. Percentage of the population surviving at day 7 is provided when applicable. Error bars indicate SE of the mean.

The groups of the tested mosquitoes were then divided and assessed by sex across the three concentrations tested. At 5% propylene glycol, there was a significant difference between the females that fed ad libitum and those that fed for 24 h (*p* < 0.0001), but not between the two groups of males (*p* = 0.348) (Figure 2a). The females that fed ad libitum had a higher daily survivorship at day 7 than the males within the same test group (*p* < 0.0001), and the same was true for the females and males that fed on propylene glycol for 24 h (*p* < 0.0001). Interestingly, we found no difference in the daily survivorship of the females that fed ad libitum and the males that fed for 24 h, but the inverse was significantly different (*p* < 0.0001). Many of these features were also valid at higher propylene glycol concentrations. At both 7.5% propylene glycol and 10% propylene glycol, females had a significantly higher survivorship than males in both the ad libitum and 24 h trials (*p* < 0.0001; Figure 2b,c). Th females that fed ad libitum had a significantly lower survivorship than those that fed for 24 h (*p* < 0.0001), as did the males at 10% propylene glycol (*p* = 0.007; Figure 2c). Surprisingly, at 7.5% propylene glycol, even the females that fed ad libitum had a higher survivorship than the males that fed for 24 h (*p* < 0.0001). While the females that fed for 24 h also had a higher survivorship than the males that fed ad libitum (*p* < 0.0001), this was less unexpected and also true at 10% propylene glycol (*p* < 0.0001). As is demonstrated in Figure 1, all the mosquitoes that fed on propylene glycol had a significantly lower daily survivorship than the control groups, which fed on 5% sucrose alone.

### 3.3. Propylene Glycol Decreases the Survivorship of Ae. albopictus and Cx. pipiens 

**Figure 3:**
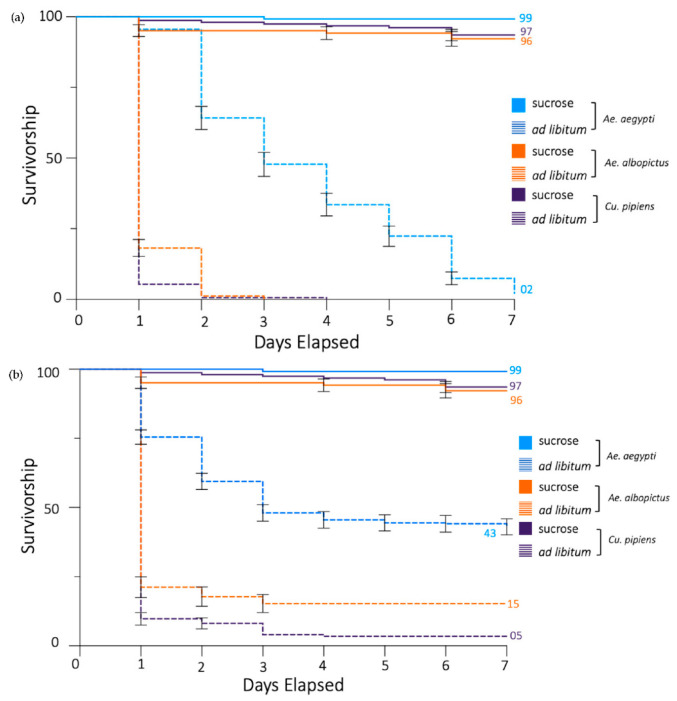
Propylene glycol reduces the daily survivorship of *Ae. aegypti, Ae. albopictus,* and *Cx. pipiens* mosquitoes. (**a**) A Kaplan–Meier curve demonstrates the decline in mosquito survivorship over the course of 7 days. Populations fed on 5% sucrose or 5% sucrose + 5% propylene glycol ad libitum. Percentage of the population surviving on day 7 is provided when applicable. Error bars indicate SE of the mean. (**b**) Populations fed on 5% sucrose ad libitum or 5% sucrose + 5% propylene glycol for 24 h, at which point it was replaced with 5% sucrose. Percentage of the population surviving at day 7 is provided when applicable. Error bars indicate SE of the mean.

The effects of propylene glycol on laboratory populations of *Ae. albopictus* and *Cx. pipiens* were also studied and compared to the effects on *Ae. aegypti*. There was no difference in the daily survivorship of the groups of each species for sucrose-only controls (Figure 3). However, in each of the species, the groups that fed ad libitum had a decreased survivorship compared with the controls (*p* < 0.0001). Interestingly, there was a difference in the daily survivorship between the species that fed on 5% propylene glycol ad libitum (Figure 3a). *Ae. aegypti* had a higher survivorship than both *Ae. albopictus* and *Cx. Pipiens* (*p* < 0.0001), and *Ae. albopictus* had a higher survivorship than *Cx. pipiens* (*p* = 0.0016).

Similar patterns emerged when comparing the groups that fed on 5% propylene glycol for 24 h (Figure 3b). In each species, the adults that had access to propylene glycol displayed a decreased daily survivorship compared to those that fed on sucrose only (*p* < 0.0001). Additionally, the differences between species observed for ad libitum trials remained. Of the groups that fed on propylene glycol for 24 h, *Ae. aegypti* again had the highest survivorship (*p* < 0.0001), and *Ae. albopictus* had a higher proportion surviving than *Cx. pipiens* (*p* = 0.0007).

## 4. Discussion

Our results indicate that propylene glycol, a GRAS compound found in numerous commercial products, reduces the daily survivorship of three predominant vector mosquito species, namely, *Ae. aegypti, Ae. albopictus*, and *Cx. pipiens*, which were selected due to their continuing impacts on public health [1,5]. Reducing the daily survivorship of adult mosquitoes can reduce the average population life expectancy, thereby decreasing vector capacity and disease transmissibility [51]. Importantly, this effect could be realized even at sub-lethal concentrations of propylene glycol. Decreasing the average life expectancy of female mosquitoes, even slightly, could lead to a significant reduction in the number of females that survive long enough to become infected and complete the extrinsic incubation period for viruses, which is typically on the order of 7–14 days [51,52,53]. This may have the further benefit of decreasing the probability of a species developing resistance mechanisms to propylene glycol, as adults will likely have already mated and reproduced before the compound affects survivorship.

We initially observed that populations of *Ae. aegypti* feeding on the compound in combination with sucrose ad libitum had a diminished survivorship at 7 days compared to groups feeding on sucrose only. As the compound was in a solution with sucrose, the mortality of the mosquitoes can be attributed to the presence of the compound and not from a lack of nutritive content. This assumption was confirmed by visually inspecting the feeding status of trial groups. Although not specifically quantified, we examined the abdomens of deceased mosquitoes and found, without exception, the presence of the blue dye, indicating that adult mosquitoes were imbibing the test solutions. Another result that supports this conclusion is that propylene glycol affected daily survivorship in a dose-dependent manner, something that would not be expected if adults were simply not feeding. 

To better model a natural setting in which mosquito populations may feed from multiple sources, we then conducted feeding trials in which the propylene glycol + sucrose solution was present in the mosquito cage for 24 h, at which point it was replaced by sucrose only [54]. Again, we observed a reduced daily survivorship in test cages compared to those that were never exposed to propylene glycol, though to a lesser extent than those feeding ad libitum. These results demonstrate that laboratory populations of *Ae. aegypti* that feed on propylene glycol have a reduced survivorship compared to those that do not. When feeding on propylene glycol ad libitum, the populations were completely eliminated at the 7.5% and 10% concentrations, and an average of only 2.24% of the population remained on the seventh day at the 5% concentration. Even when we presented the compound to the groups for 24 h, daily survivorship was reduced to below 50% at all concentrations (Figure 1, Figure 2 and Figure 3). This demonstrates a potent effect of propylene glycol on *Ae. Aegypti* adults. Interestingly, when the effect of propylene glycol on *Ae. aegypti* was assessed according to sex, we found that males had a significantly lower daily survivorship than females under both feeding paradigms (Figure 2). We propose that this effect is due to behavioral differences in males and females [55,56]. While female mosquitoes tended to rest on the surfaces of the cages, males flew continuously within the enclosure, thus increasing the frequency of sugar meal consumption and, therefore, the amount of propylene glycol ingested. Another factor could be adult size. Although not quantified in our study, adult males tended to be smaller than females. Thus, the effective dose of propylene glycol per milligram of body weight may have been higher in males.

We continued this study by testing the effects of propylene glycol on laboratory populations of *Ae. albopictus* and *Cx. pipiens*. The consumption of propylene glycol imposed a much greater survivorship-reducing effect on both species when compared to *Ae. aegypti* under both feeding paradigms. Interestingly, this effect was most pronounced in *Cx. pipiens*, although the underlying reason for this difference is unknown. It would be interesting to examine the fate of propylene glycol in the digestive tract and hemolymph of these species to assess potential differences in enzymatic digestion or biochemical transformation, which produces variability in outcomes.

Another important factor that remains to be investigated is the potential off-target effects of propylene glycol. This study was limited to the effects of propylene glycol on daily survivorship in adult mosquitoes, but it will be necessary to consider the broader impacts on other arthropods, and perhaps vertebrate species [57,58]. However, utilizing this compound in an ATSB system in conjunction with a specific attractant and physical barriers could alleviate this issue [59]. Further research should also be conducted to determine the relevance of this substance in an ATSB context to human health. Lastly, there are many more GRAS compounds that remain to be tested for toxicity to mosquitoes, one or more of which may prove to be even more safe and effective [60].

From our results, we conclude that propylene glycol reduces the daily survivorship of adult vector mosquitoes. By extension, we propose that propylene glycol would affect the average life expectancy of vector populations in the context of ATSBs. As the observed increase in insecticide resistance in mosquitoes is an issue of global concern, the need for new and innovative management strategies is clear. Our study demonstrates that propylene glycol may represent one new compound that can be utilized for localized population reduction and the interruption of disease transmission cycles. 

Future experiments will include adding a mosquito attractant to the solution and performing field studies to determine how well propylene glycol works as an ATSB component. As our initial study demonstrates that propylene glycol leads to significant decreases in survivorship in three mosquito species in a lab setting, we conclude that propylene glycol may decrease the average life expectancy in natural populations. As such, our study provides a foundation for future ATSB design and field-based population dynamics studies, where incorporating an effective substance could reduce vector mosquito population while also ameliorating the effects on non-target populations.

## Data Availability

The data presented in this study are available in File S1: Experimental data.

## Supplementary Materials

The following supporting information can be downloaded at: https://www.mdpi.com/article/10.3390/insects13070595/s1, Figure S1: Experimental design; File S1: Experimental data.
