# Supplementary material for: Survivorship-Reducing Effect of Propylene Glycol on Vector Mosquito Populations and Its Potential Use in Attractive Toxic Sugar Baits
Source: Insects. 2022 Jun 29;13(7):595. doi: 10.3390/insects13070595 (PMC9324135; doi:10.3390/insects13070595)
Supplement: Supplementary file 1 [file insects-13-00595-s001.zip › insects-1768557-supplementary.pdf]

Figure S1: Experimental Design

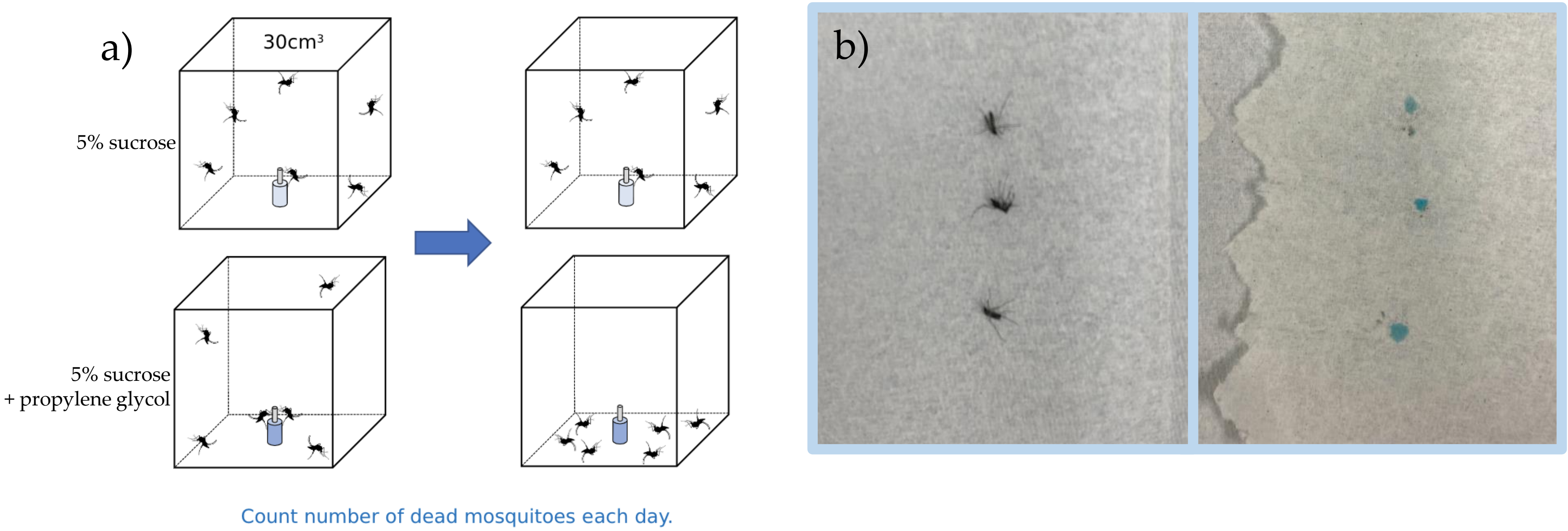

Figure S1: a) A representation of the feeding assay design b) Blue dye is used to determine feeding status of expired mosquitoes. Three deceased female *Ae. aegypti* on a paper towel (left). Blue dots appear after pressing on carcasses with another paper towel reveals that all three mosquitoes had fed on the compound (right).
